# Perspectives on Thoracic Endovascular Aortic Repair for Uncomplicated Type B Aortic Dissection: An International Survey Within the SUNDAY Network

**DOI:** 10.3390/jcm15197604

**Published:** 2026-09-30

**Authors:** Bianca Biersteker, Felix Hers, Meike Langenhuijzen, Jorg Lucas de Bruin, Jean-Paul Petrus Maria de Vries, Mark Dirven, Martin Teraa, Kevin Mani, Timothy Andrew Resch, Barend Mathijs Eduard Mees, Kak Khee Yeung, Jacob Budtz-Lilly, Joost Renier van der Vorst

**Affiliations:** 1 Department of Vascular Surgery, Leiden University Medical Center, Albinusdreef 2, 2333 ZA Leiden, The Netherlands; m.langenhuijzen@lumc.nl (M.L.); j.r.van_der_vorst@lumc.nl (J.R.v.d.V.); 2 Department of Vascular Surgery, Maastricht University Medical Center, P. Debyelaan 25, 6229 HX Maastricht, The Netherlands; f.hers@dica.nl (F.H.); barend.mees@mumc.nl (B.M.E.M.); 3 Dutch Institute for Clinical Auditing, Rijnsburgerweg 10, 2333 AA Leiden, The Netherlands; 4 Department of Vascular Surgery, Erasmus Medical Center, Molenwaterplein 40, 3015 GB Rotterdam, The Netherlands; j.debruin@erasmusmc.nl; 5 Department of Surgery, Division of Vascular Surgery, University Medical Center Groningen, University of Groningen, Hanzeplein 1, 9713 GZ Groningen, The Netherlands; j.p.p.m.de.vries@umcg.nl; 6 Department of Vascular Surgery, Radboudumc, Geert Grooteplein Zuid 10, 6525 GA Nijmegen, The Netherlands; mark.dirven@radboudumc.nl; 7 Department of Vascular Surgery, University Medical Center Utrecht, Hiedelberglaan 100, 3584 CX Utrecht, The Netherlands; m.teraa@umcutrecht.nl; 8 Department of Surgical Sciences, Uppsala University, Sjukhusvägen 70, 751 85 Uppsala, Sweden; kevin.mani@uu.se; 9 Department of Vascular Surgery, Copenhagen University Hospital Rigshospitalet, Blegdamsvej 9, 2100 Copenhagen, Denmark; timothy.andrew.resch@regionh.dk; 10 Faculty of Health Sciences, Copenhagen University, Blegdamsvej 3B, 2200 Copenhagen, Denmark; 11 Department of Vascular Surgery, Amsterdam University Medical Centers, Location AMC, Meibergdreef 9, 1105 AZ Amsterdam, The Netherlands; k.yeung@amsterdamumc.nl; 12 Department of Atherosclerosis & Aortic Diseases, Amsterdam Cardiovascular Sciences, Amsterdam University Medical Centers, Location AMC, Meibergdreef 9, 1105 AZ Amsterdam, The Netherlands; 13 ^3^ Department of Vascular Surgery, Aarhus University Hospital, Palle Juul-Jensens Boulevard 99, DK-8200 Aarhus, Denmark; jacoblilly@clin.au.dk

**Keywords:** type B aortic dissection, uncomplicated, TEVAR, survey, clinical practice, treatment strategies

## Abstract

**Background**: Thoracic endovascular aortic repair (TEVAR) is increasingly considered in selected patients with uncomplicated type B aortic dissection (uTBAD), but uncertainty remains regarding indications for intervention and treatment strategies. This study aimed to explore potential variation in TEVAR indications and treatment strategies for uTBAD among principal investigators (PIs) of the Scandinavian trial of Uncomplicated Aortic Dissection Therapy (SUNDAY). **Methods**: A prospective, cross-sectional, questionnaire-based study was conducted among PIs of the SUNDAY trial network. A structured survey was developed by the first and last author using contemporary guidelines and relevant literature and was subsequently reviewed by all co-authors. The questionnaire addressed three topics: definitions, TEVAR indications, and treatment strategies. Data were collected via Castor and analyzed using SPSS version 29.0. A center-weighted sensitivity analysis and within-center concordance analysis were performed to account for multiple respondents from the same center. **Results**: The response rate was 78.2% (43/55 PIs), representing 31 centers. The presence or development of complications was the main driver for TEVAR (37/43, 86.0%). Rupture (43/43, 100%), clinical signs of malperfusion (43/43, 100%), and rapid aortic expansion (38/43, 88.4%) were consistently considered indications for TEVAR, whereas more variation was observed for refractory pain (28/43, 65.1%) and refractory hypertension (26/43, 60.5%). Interpretation of high-risk imaging features varied, with 37.2% (16/43) considering multiple features an indication for TEVAR. Procedural strategies varied, with spot stenting selected by 58.1% (25/43) of respondents, although more extensive aortic coverage was also reported. Landing zone characteristics and oversizing preferences were more consistent, with 79.1% (34/43) defining a healthy landing zone by the absence of intramural hematoma or dissection, 58.1% (25/43) preferring a landing zone length of 2 cm, and 51.2% (22/43) preferring 10% stent graft oversizing. Center-weighted sensitivity analyses showed generally similar estimates, whereas within-center concordance varied across items (range 20–100%), with complete agreement for 8 of 50 items. **Conclusions**: Among SUNDAY PIs, there was broad agreement regarding the distinction between uncomplicated and complicated type B aortic dissection (TBAD) and the role of TEVAR in the presence of complications, whereas variation remained in the interpretation of high-risk imaging features and procedural strategies. These findings highlight variation in decision-making regarding the management of uTBAD within the SUNDAY network and support the need for prospective evidence to guide TEVAR decision-making.

## 1. Introduction

Type B aortic dissection (TBAD) has an incidence of approximately 3.9–6.0 per 100,000 persons per year [1,2,3,4], with approximately 50–60% classified as uncomplicated [5]. Prompt medical therapy, including anti-impulse therapy and adequate analgesia, is recommended as initial treatment for acute TBAD (<14 days after diagnosis) [5,6,7,8,9]. Thoracic endovascular aortic repair (TEVAR) is the standard of care for complicated TBAD (cTBAD) [5,6,7,8,9], and is increasingly used in the management of uncomplicated TBAD (uTBAD), although routine use outside clinical trials is not recommended [5,6,7,8,9]. Randomized trials, including ADSORB and INSTEAD, have demonstrated potential benefits of TEVAR in uTBAD, particularly with regard to aortic remodeling [10,11,12]. Early TEVAR during the subacute phase (14–90 days after diagnosis) may be considered in selected patients [5,6,7,9].

Current international guidelines differ in their recommendations for TEVAR in uTBAD. Several guidelines identify high-risk imaging features associated with adverse aortic outcomes, and recommend considering TEVAR in patients presenting with these features (Class IIa-b, Level B-NR, B-R and C) [6,9,13]. However, the evidentiary support for individual high-risk imaging features varies, and many proposed features are supported predominantly by observational rather than randomized evidence [5,7,9,13,14]. In contrast, the latest European Society for Vascular Surgery (ESVS) guidelines recommend considering TEVAR in selected patients with uTBAD and identify an initial aortic diameter >40 mm as the imaging parameter most consistently associated with subsequent aneurysmal degeneration, while early rapid expansion is included as an additional factor supporting consideration of TEVAR (Class IIb, Level C), despite limitations in its definition and validation [5].

All guidelines agree on the clinical indications for TEVAR, including rupture, refractory pain or hypertension and clinical signs of malperfusion [5,6,7,9,13]. However, definitions of these complications remain inconsistent. For example, definitions of refractory pain and hypertension vary, with differences in treatment duration and thresholds, and in some cases the absence of clearly defined criteria [5,6,7,9,13].

Guidance on procedural strategy is limited, as current guidelines do not consistently address landing zone characteristics, stent graft configuration, or adjunctive procedures, leaving room for clinical judgment [5,6,7,9,13].

The SUNDAY trial is currently investigating whether TEVAR impacts five-year survival in patients with uTBAD by randomizing participants to either standard medical therapy (SMT) alone or SMT with TEVAR during the subacute phase [3]. Given limited and heterogeneous guidance on TEVAR in uTBAD, the pragmatic SUNDAY trial design allows participating centers to follow their established local practice regarding medical and endovascular treatment. This leaves room for clinical judgment and inter-center variation. A previous questionnaire among SUNDAY trial investigators identified variation in several aspects of medical management of uTBAD, particularly in the definitions of refractory hypertension and pain, and in the classification of dissection chronicity [15]. Beyond this SUNDAY-specific survey, previous international surveys have focused primarily on TEVAR decision-making in uTBAD [16,17,18]. We therefore conducted a questionnaire-based study to investigate potential variations in both TEVAR indications and treatment strategies for uTBAD among PIs of the SUNDAY trial. This provides insight into current practice patterns relevant to the interpretation and generalizability of the trial.

## 2. Materials and Methods

### 2.1. Study Design

This was a prospective, cross-sectional, questionnaire-based study. A structured survey was distributed via Castor EDC (Castor, Amsterdam, The Netherlands), a secure web-based electronic data capture platform. Completion of the questionnaire implied informed consent. Data were collected over a six-week period from late January through February 2026, with three reminder emails sent to non-responders. The study is reported in accordance with the Consensus-Based Checklist for Reporting of Survey Studies (CROSS) [19]. The completed checklist is provided as Supplementary Material S1.

### 2.2. Study Population

All PIs from the SUNDAY trial network were invited to participate. Compared with the previous questionnaire by Saricilar et al. [15], the SUNDAY trial network had expanded by the time of the present study, resulting in an increase in the number of potential participants from 46 to 55. Thus, 55 PIs were approached, with some centers represented by more than one PI. Because respondent-level data from the previous questionnaire [15] were not available for comparison, the number of respondents participating in both questionnaires could not be determined. A list of SUNDAY collaborators is provided as Supplementary Material S2.

### 2.3. Questionnaire Design

The questionnaire was developed by the first and last authors and subsequently reviewed by all co-authors in three rounds. Questionnaire content was based on contemporary international guidelines and the relevant literature on uTBAD, with particular attention to areas in which recommendations are heterogeneous, insufficiently defined, or allow room for clinical interpretation [5,6,7,9,13,20,21]. During the review process, the authors assessed the relevance and clarity of questionnaire items, with additional items included based on their relevance to the study objectives and consensus among the authors. Prior to distribution, the questionnaire was tested for technical functionality and correct routing within Castor.

Three main topics were addressed: definitions, indications for TEVAR, and treatment strategies for TEVAR, with a focus on the subacute phase. For clinical indications such as refractory hypertension and pain, the questionnaire specifically assessed when respondents considered these conditions sufficiently refractory to warrant TEVAR, as well as the criteria used to define them. Questions were primarily closed-ended and collected categorical data, with some including free-text or numerical responses. Some questionnaire items allowed multiple responses, and these were analyzed as multiple-response items. A copy of the questionnaire is provided as Supplementary Material S3.

### 2.4. Data Collection and Statistical Analysis

Data were collected in Castor using personalized links, allowing for direct data entry and single submission. Records were de-identified, with country retained as the sole identifying variable for descriptive purposes. All questionnaire items were mandatory, and no unanswered items were recorded. Some items were conditionally displayed based on respondents’ previous answers.

Data were analyzed using SPSS version 29.0 (IBM, Armonk, NY, USA). Categorical variables were summarized as frequencies and percentages. Numerical variables were reported as mean (standard deviation) or median (interquartile range), as appropriate, or categorized into frequencies and percentages. Free-text responses, excluding numerical data, were either categorized and summarized as frequencies, or described. Given the descriptive aim and sample size, no formal between-country comparisons were made. To account for the potential influence of multiple respondents from the same center, center-weighted sensitivity and within-center concordance analyses were performed. For the center-weighted sensitivity analysis, the proportion of respondents selecting each response option was calculated separately for each center, after which the center-specific proportions were averaged across participating centers, giving each center equal weight irrespective of the number of responding PIs. Within-center concordance was assessed among the 12 centers with two responding PIs and was defined as the proportion of these centers in which both respondents provided the same response to a given questionnaire item.

## 3. Results

### 3.1. Demographics

**Figure 1:**
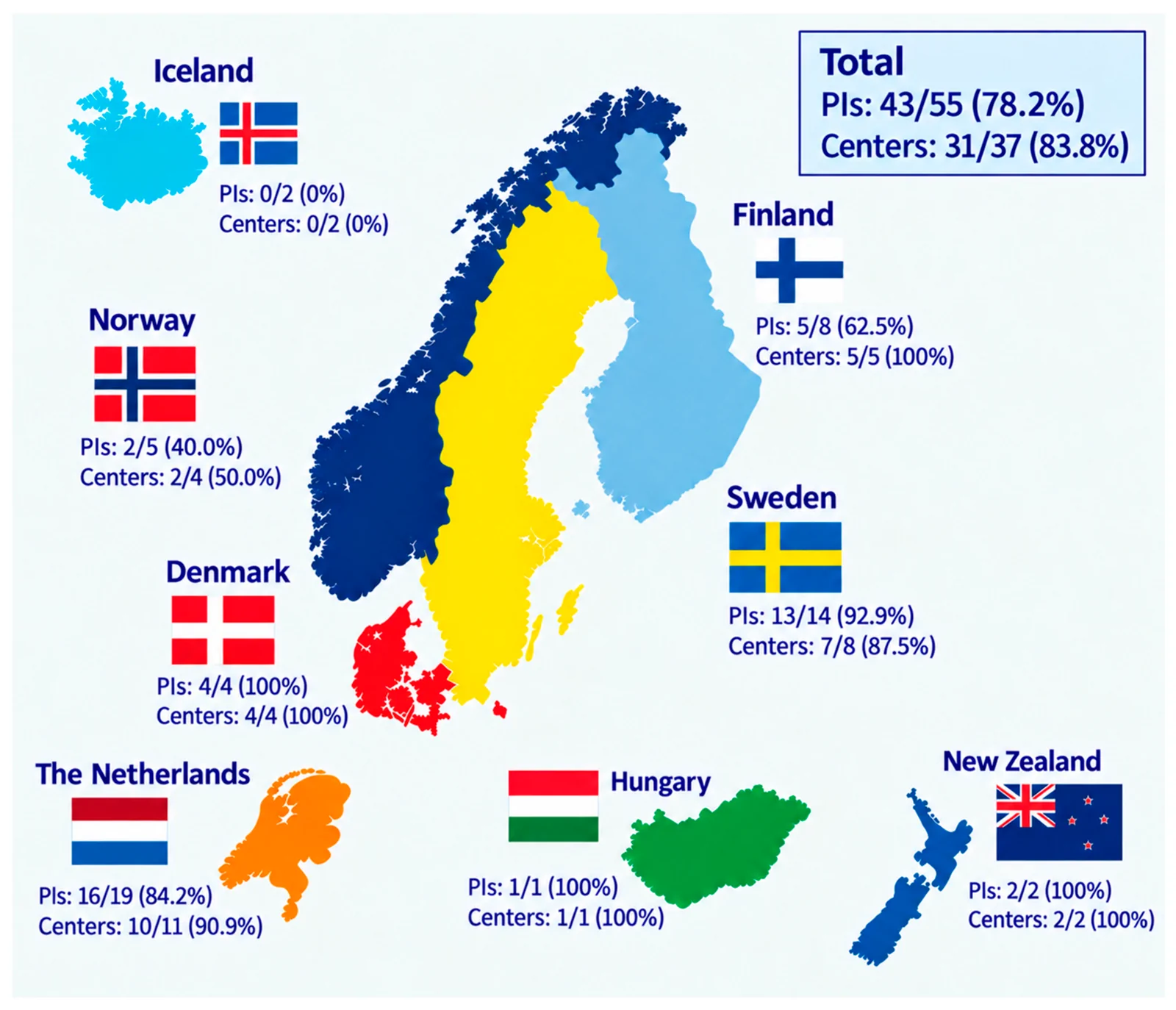
Regional distribution of invited and responding PIs and participating centers, by country.

A total of 43 of 55 PIs (78.2%) responded during the data collection period, representing 31 participating centers across seven countries. Most responses originated from the Netherlands (16 of 19 invited PIs, representing 10 of 11 invited centers) and Sweden (13 of 14 invited PIs, representing 7 of 8 invited centers), whereas other countries were represented by one to five respondents; no responses were received from Iceland (Figure 1; Table 1).

Most respondents were vascular surgeons (40/43, 93%), followed by interventional radiologists (2/43, 4.7%) and cardiac surgeons (1/43, 2.3%) (Table 1). Mean years of working experience was 17.4, and the median annual number of treated uTBAD cases was 15.

### 3.2. Definitions

**Figure 2:**
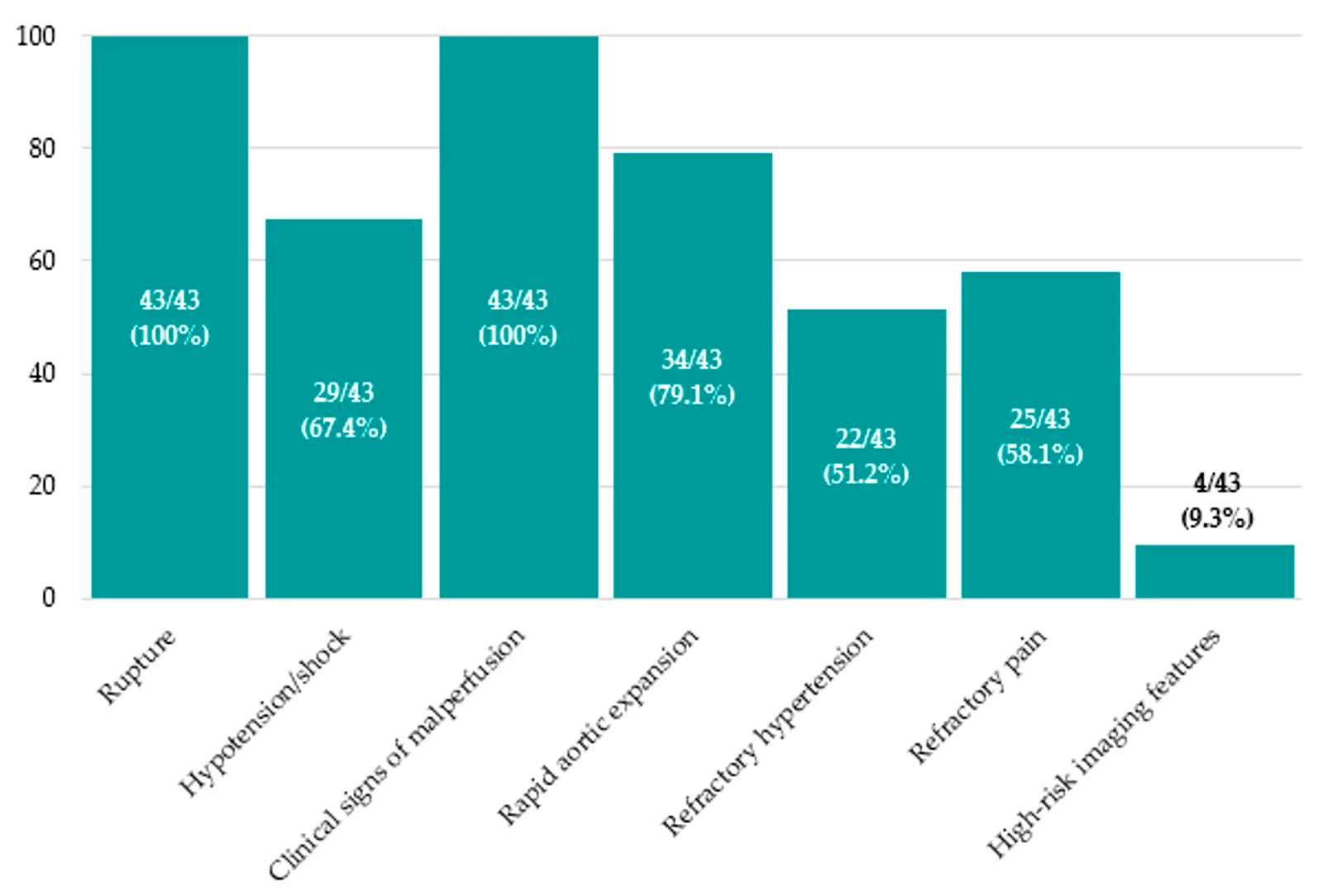
Percentage of respondents who considered type B aortic dissection (TBAD) complicated in different cases (%). Multiple responses were allowed; percentages therefore do not necessarily sum to 100%.

The criteria used to distinguish uncomplicated from complicated TBAD are shown in Figure 2. All respondents considered TBAD to be complicated in the event of rupture or clinical signs of malperfusion. Greater variation was observed for refractory pain (25/43, 58.1%) and refractory hypertension (22/43, 51.2%), with only 9.3% (4/43) of respondents considering high-risk imaging features sufficient to classify TBAD as complicated.

Table 2 summarizes the perceived definition of rapid aortic expansion, demonstrating variation in the reported responses. A growth of >10 mm between serial imaging was the most frequently reported definition in both the acute and subacute phases (25/43, 58.1% and 22/43, 51.2% respectively).

### 3.3. Indications for TEVAR

#### 3.3.1. High-Risk Imaging Features

**Figure 3:**
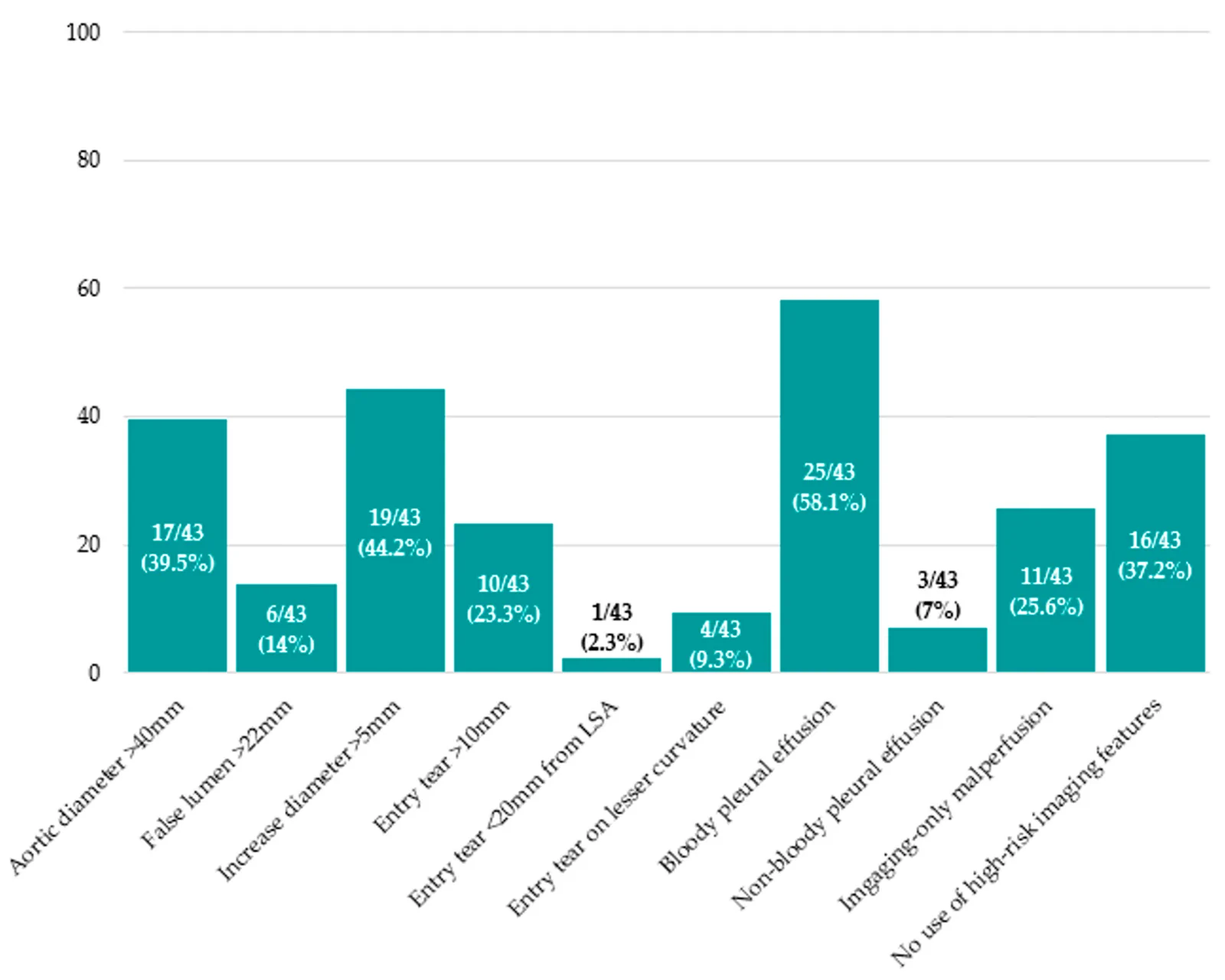
Perceived thoracic endovascular aortic repair (TEVAR) indications based on high-risk imaging features in both acute and subacute uTBAD (in %). Multiple responses were allowed; percentages therefore do not necessarily sum to 100%. LSA = Left subclavian artery.

Variation was observed regarding the consideration of high-risk imaging features as indications for TEVAR in uTBAD in both the acute and subacute phase (Figure 3). Overall, 16 of 43 (37.2%) respondents did not consider high-risk imaging features as an indication for TEVAR. Among individual imaging features, bloody pleural effusion was selected most often (25/43, 58.1%), while no single feature showed consistent selection among respondents.

#### 3.3.2. Clinical Assessment

**Figure 4:**
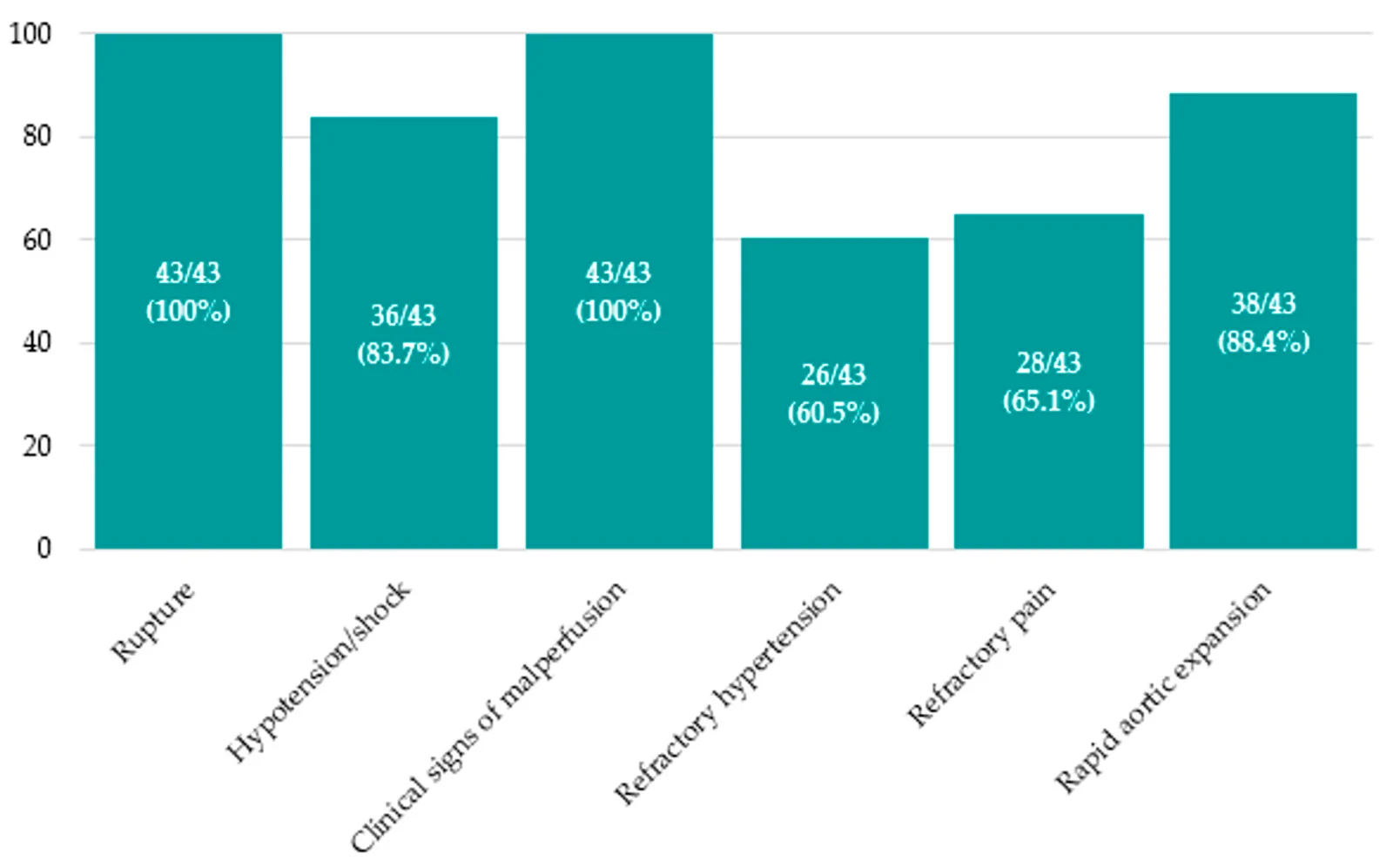
Perceived TEVAR indications based on clinical complications in TBAD (in %). Multiple responses were allowed; percentages therefore do not necessarily sum to 100%.

All respondents would consider TEVAR in cases of rupture or clinical signs of malperfusion, and 88.4% (38/43) in case of rapid aortic expansion (Figure 4). Refractory hypertension and pain were considered indications for TEVAR by 60.5% (26/43) and 65.1% (28/43) of respondents, respectively.

Among the 26 respondents (60.5% of all respondents) considering refractory hypertension an indication for TEVAR, 23.1% (6/26) defined it as failure to achieve target blood pressure despite maximal antihypertensive treatment for 72 h, and 38.5% (10/26) for more than one week. Additionally, 30.8% (8/26) would consider TEVAR if more than five antihypertensive agents were required, 26.9% (7/26) if more than three agents were needed, and 30.8% (8/26) did not consider the number of antihypertensive agents in their decision-making. For refractory pain (*n* = 28, 65.1% of all respondents), 32.1% (9/28) would consider TEVAR if pain persisted for >48 h despite maximal treatment, whereas 28.6% (8/28) would wait more than one week.

Among respondents, 86% (37/43) would consider TEVAR in the presence of clinical complications, 37.2% (16/43) in the presence of multiple high-risk imaging features, and 20.9% (9/43) would not consider TEVAR in uTBAD.

### 3.4. TEVAR Treatment

#### 3.4.1. Stenting Strategies in TEVAR

**Figure 5:**
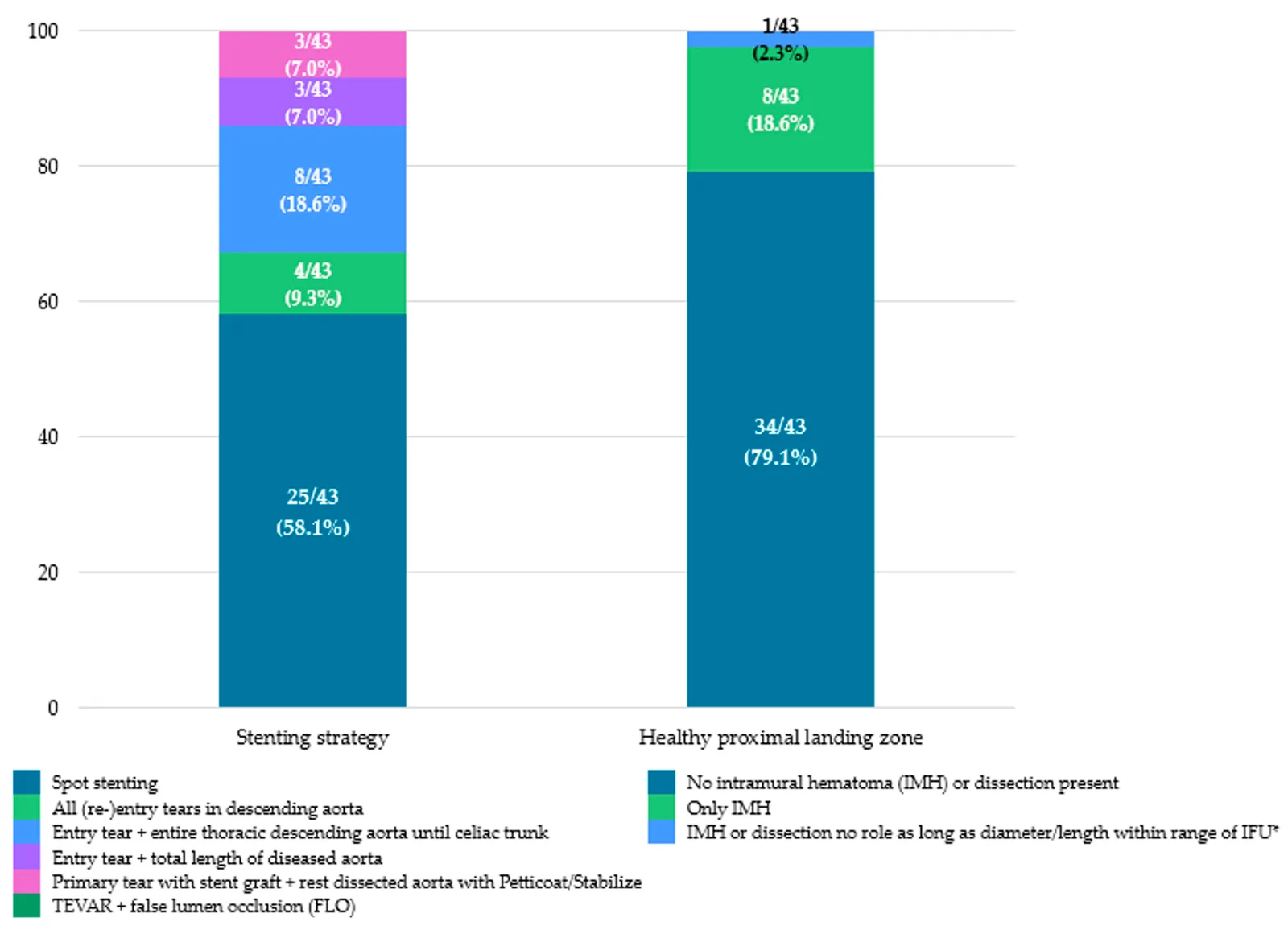
Preferred TEVAR stenting strategy and definition of a healthy proximal landing zone in uTBAD (in %). * IFU = instructions for use.

Variation was observed in the preferred stenting strategies (Figure 5), with 58.1% (25/43) preferring spot stenting. A healthy proximal landing zone was defined as a segment free from both intramural hematoma (IMH) and dissection by 34 of 43 (79.1%) respondents (Figure 5). A healthy landing zone length of 2 cm was preferred by 58.1% (25/43) of respondents, while 12 of 43 (27.9%) respondents preferred 3 cm. For the minimum required healthy landing zone length, 2 cm was selected by 27 of 43 (62.8%) respondents and 1 cm by 30.2% (13/43).

A total of 51.2% (22/43) preferred 10% oversizing, followed by 23.3% (10/43) preferring 5% and 9.3% (4/43) preferring 15%. Proximal diameter sizing would be based on the intended proximal sealing zone, regardless of the presence of pathology, according to 53.5% (23/43). For primary entry tears located in aortic zones 4 or 5, 25 of 43 (58.1%) respondents reported extending coverage to the level of the left subclavian artery (LSA) take-off only in the presence of proximal pathology, whereas 32.6% (14/43) would limit coverage to the primary entry tear.

#### 3.4.2. LSA Revascularization

**Figure 6:**
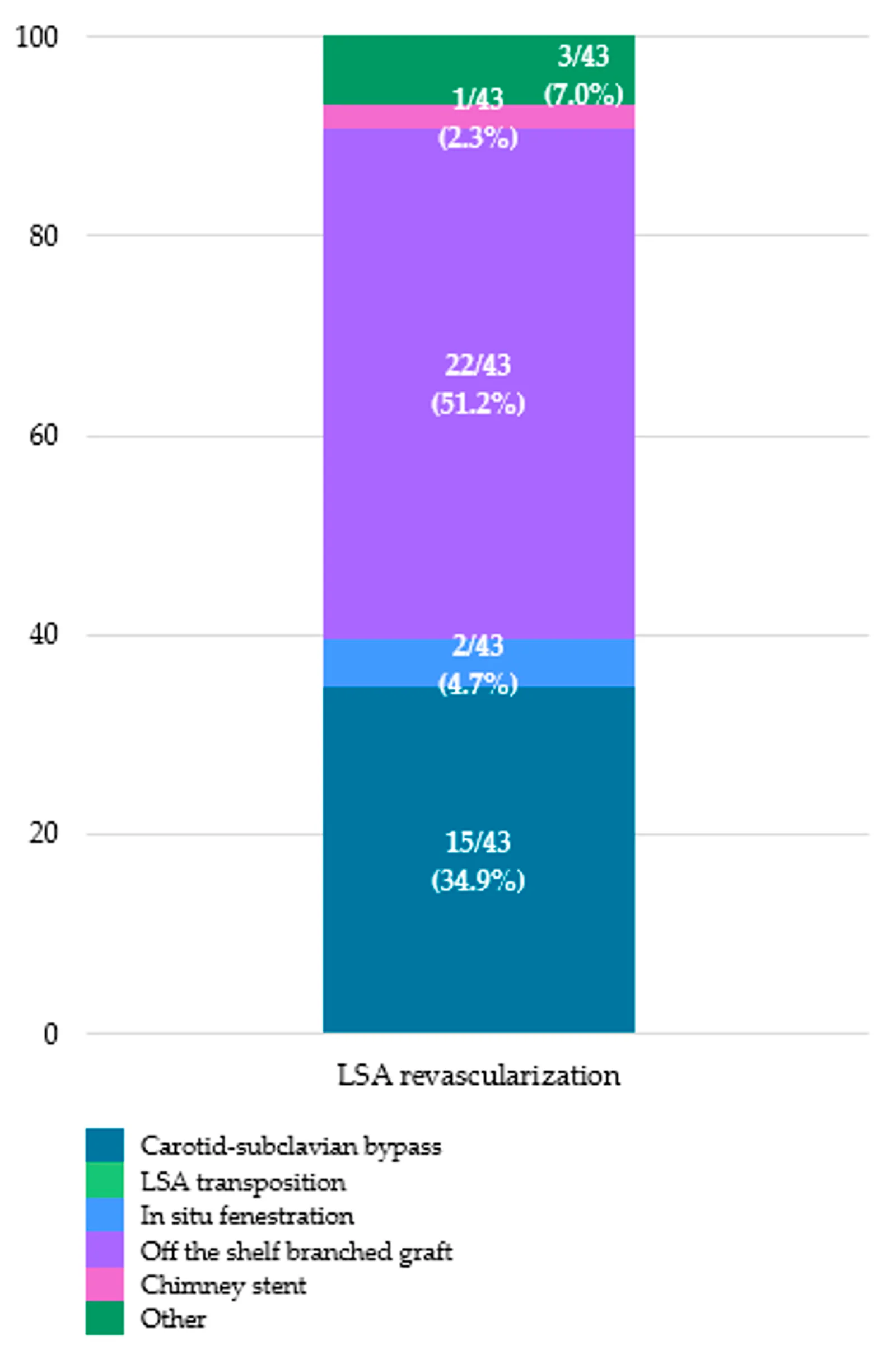
Preferred techniques for LSA revascularization (in %).

In elective uTBAD cases requiring LSA coverage, variation was observed in decision-making regarding revascularization. Preventive revascularization was selected by 58.1% (25/43) of respondents, whereas 27.9% (12/43) would base this decision on anatomic features. Features mentioned by more than one respondent were a dominant left vertebral artery (*n* = 4) and circle of Willis patency (*n* = 5); other single responses included vertebral artery patency and previous coronary artery bypass grafting (CABG) with a left mammary artery graft. Some respondents based the decision on clinical context, considering LSA revascularization in the acute setting only if symptoms developed. Preferred LSA revascularization techniques are summarized in Figure 6, with off-the-shelf branched grafts and carotid-subclavian bypass most commonly selected.

#### 3.4.3. Cerebrospinal Fluid (CSF) Drainage

CSF drainage was predominantly considered a rescue intervention following TEVAR-related neurologic symptoms by 37 of 43 (86%) respondents. Other indications, including extensive aortic coverage (>20 cm) (9/43, 20.9%) and previous abdominal aortic aneurysm (AAA) repair (3/43, 7%), were selected less frequently.

#### 3.4.4. Access Routes and Anesthesia for TEVAR

General anesthesia was preferred by 36 of 43 (83.7%) respondents. Percutaneous femoral access was the preferred access route for TEVAR for 40 of 43 (93%) respondents.

**Figure 7:**
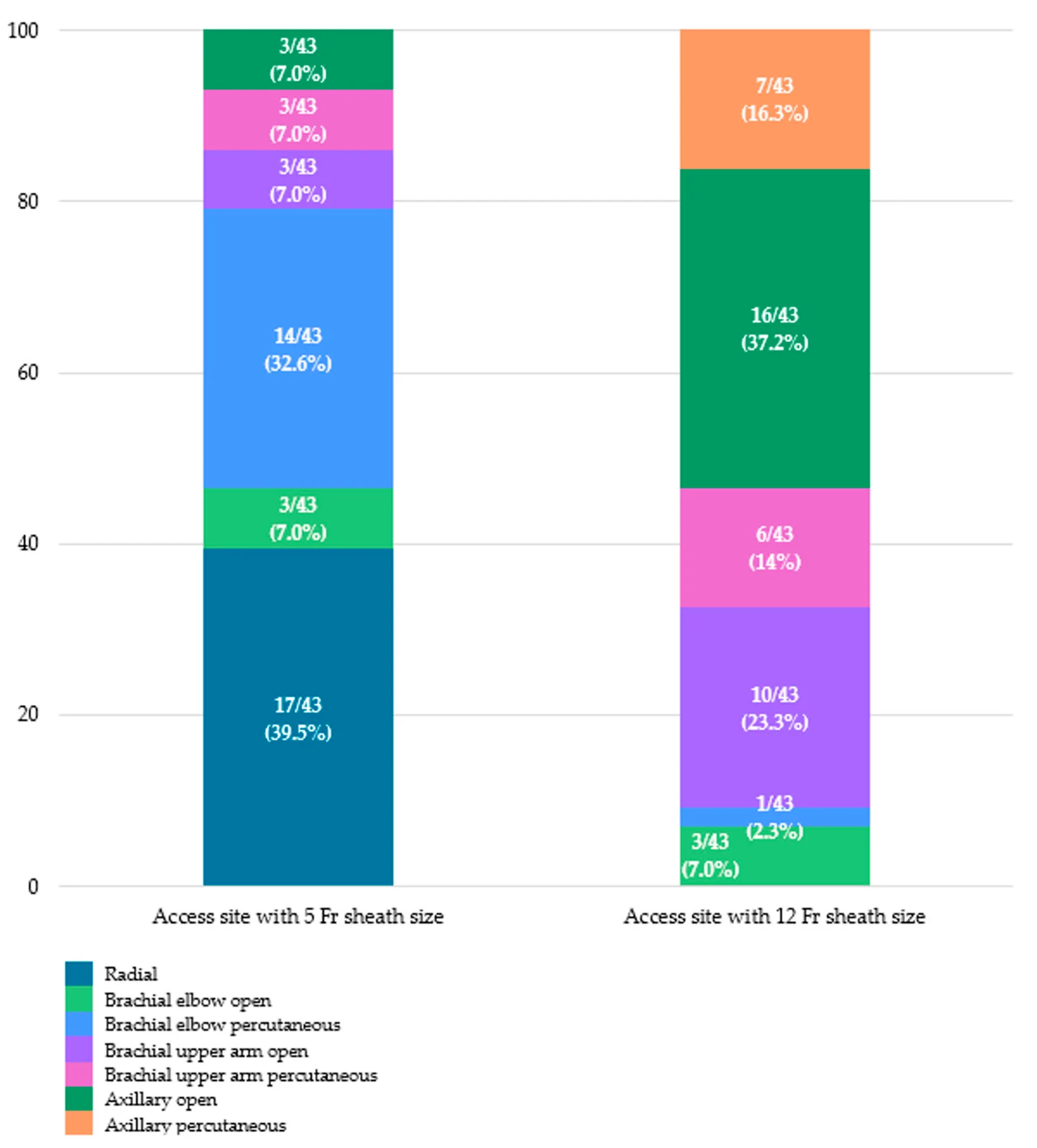
Preferred upper extremity access routes (in %).

Variation was observed for upper extremity access. For a 5 Fr sheath, radial access (17/43, 39.5%) and percutaneous brachial access at the elbow (14/43, 32.6%) were most frequently selected. For a 12 Fr sheath, open axillary access (16/43, 37.2%) and open brachial access in the upper arm (10/43, 23.3%) were the most selected access sites (Figure 7).

### 3.5. Center-Weighted Sensitivity and Within-Center Concordance Analyses

Center-weighted sensitivity analyses accounting for multiple respondents from the same center showed generally similar estimates to the respondent-level analyses. Across the assessed response items, the absolute differences between respondent-level and center-weighted estimates were generally small, with a maximum difference of 10.3 percentage points. Within-center concordance varied across the 57 assessed response items, ranging from 20% to 100%. Among the 50 response items assessed across all 12 centers with two responding PIs, complete concordance was observed for 8 items. Full results of the center-weighted sensitivity and within-center concordance analyses are provided in Supplementary Material S4.

## 4. Discussion

This international survey investigated potential variations in TEVAR indications and treatment strategies for uTBAD among PIs of the SUNDAY trial. While some aspects of decision-making were consistent, substantial uncertainty remains regarding the interpretation of high-risk features and several aspects of TEVAR strategy. Although randomized trials have demonstrated potential benefits of TEVAR in uTBAD, particularly with regard to aortic remodeling [10,11,12], evidence remains insufficient to establish whether these potential benefits translate into improved clinical outcomes and which patients are most likely to benefit from TEVAR. In addition, evidence is limited regarding several aspects of TEVAR strategy, including procedural strategies and the use of adjunctive techniques. Given these uncertainties and the lack of clear guidance, variation in reported clinical decision-making was expected.

Areas with relatively consistent responses mainly concerned the criteria used to distinguish uncomplicated from complicated TBAD, as well as procedural aspects supported by established recommendations. Respondents consistently classified TBAD as complicated in the presence of rupture, clinical signs of malperfusion, or rapid aortic expansion, consistent with current guideline definitions [5]. Rupture and clinical signs of malperfusion were also consistently considered indications for TEVAR, in accordance with current guideline recommendations [5]. Furthermore, current guidelines consider early aortic expansion in selected patients with otherwise uncomplicated acute TBAD and suitable anatomy as a potential indication for TEVAR, preferably during the subacute phase [5]. In this survey, most respondents regarded rapid aortic expansion as an indication for TEVAR. Similarly, preferences regarding the definition of a healthy proximal landing zone, landing zone length, and stent graft oversizing were generally aligned with guideline recommendations and instructions for use [5].

In contrast, greater variation was observed in clinical scenarios where evidence is limited, and treatment thresholds remain less clearly defined. Although refractory hypertension and pain were considered indications for TEVAR by a proportion of respondents, no clear predominance was observed. Furthermore, variation existed in the thresholds respondents used to consider refractory hypertension and pain indications for TEVAR, including treatment duration and the number of antihypertensive agents required. These findings highlight the lack of clear guidance on the definition and clinical application of these features in uTBAD. These findings are consistent with previous observations by Saricilar et al., who reported variation in the definitions of refractory hypertension and pain [15]. Although guidelines suggest criteria such as the use of multiple antihypertensive classes, specific thresholds and treatment durations remain inconsistently defined [5,6,9]. In this survey, refractory hypertension referred to hypertension considered sufficiently unresponsive to medical treatment to warrant TEVAR and was not defined according to other definitions of resistant hypertension. Similarly, although rapid aortic expansion was consistently regarded as a potential indication for TEVAR, variation was observed in its definition regarding both threshold and disease phase. In addition, a >5 mm increase between serial imaging as a high-risk imaging feature was less consistently considered an indication for intervention. This apparent discrepancy may reflect differences in the clinical interpretation of rapid aortic expansion and predefined imaging criteria.

Uncertainty remained regarding the role of high-risk imaging features in guiding TEVAR indications. Importantly, high-risk imaging features should be distinguished from clinical complications that define complicated TBAD. Individual features were not consistently considered indications for intervention. In particular, an initial aortic diameter >40 mm was not uniformly regarded as an indication for intervention, despite being highlighted in current ESVS guidelines as a factor supporting consideration of TEVAR [5]. Although an initial aortic diameter >40 mm is among the imaging features most consistently associated with subsequent aneurysmal degeneration, the evidence supporting its use to guide pre-emptive TEVAR remains limited [5]. Similarly, early aortic expansion is considered a potential factor supporting TEVAR, but its definition and clinical relevance remain insufficiently established [5]. Other guidelines describe a broader range of high-risk imaging features, although the evidence supporting these criteria remains limited [5,6,7,9,13]. The lack of prospective validation may contribute to variability in their interpretation and clinical application.

Procedural strategies and adjunctive techniques showed further variation. No single stenting strategy predominated, with spot stenting selected by 58.1% of respondents, while more extensive aortic coverage was also reported. More extensive coverage may improve aortic remodeling, but may also increase the risk of spinal cord ischemia (SCI), highlighting the challenge of balancing effective dissection management against procedure-related complications [22,23,24]. Variation was observed in LSA management, both regarding the decision to perform revascularization and the preferred revascularization strategy. Current ESVS guidelines recommend considering LSA revascularization in patients undergoing elective TEVAR with intentional LSA coverage (Class IIa, Level B), while the choice between open and endovascular revascularization should be individualized (Class IIa, Level C) [5,20]. Contemporary evidence, largely based on observational studies, has associated LSA revascularization with lower rates of stroke and spinal cord ischemia [21]. In our survey, 51.2% of respondents preferred an off-the-shelf branched graft. Recent studies have demonstrated the feasibility of fully endovascular zone 2 TEVAR using single-branched devices in selected patients [25,26]. These studies, however, primarily demonstrate technical feasibility, which does not in itself define the appropriate indication for these devices.

The observed variability in areas of clinical uncertainty highlights the need for prospective evidence to guide the management of uTBAD. Importantly, this variation was observed among PIs participating in the same international randomized trial, underscoring the variability in reported TEVAR preferences even within a collaborative trial network. Current guidelines provide limited recommendations regarding the role of TEVAR in uTBAD, and the clinical relevance of proposed high-risk imaging features remains uncertain. Ongoing trials, including SUNDAY, IMPROVE-AD, and EARNEST, are therefore expected to play a key role in addressing these uncertainties. These trials may help determine whether TEVAR improves long-term outcomes in patients with uTBAD and additionally provide insights into which patient subgroups may benefit from TEVAR and the relevance of proposed high-risk imaging features. The heterogeneity in reported treatment preferences observed in this survey supports the rationale behind the SUNDAY trial design. The trial was deliberately developed as a pragmatic trial evaluating TEVAR as performed in contemporary clinical practice, while allowing operators to apply established local approaches rather than mandating a uniform procedural strategy. At the same time, variation in stent coverage, oversizing, LSA management, and adjunctive techniques may introduce heterogeneity in both procedural outcomes and treatment-related risks, which should be considered when interpreting the trial results. Importantly, the SUNDAY trial prospectively captures several procedure-specific variables that may help characterize this procedural heterogeneity, including estimated aortic coverage, proximal and distal landing zones, stent graft and proximal seal diameters, and adjunctive procedures such as LSA revascularization and CSF drainage. This procedural variability is inherent to the pragmatic, strategy-based design of SUNDAY, which evaluates TEVAR as a treatment strategy rather than a specific technical approach. As such, SUNDAY aims to determine the clinical value of TEVAR in uTBAD while accounting for the variability inherent in current treatment strategies.

### Limitations

Questionnaire-based research is inherently associated with several limitations. First, response bias may have influenced the findings, as participants’ answers can be affected by personal perspectives, experiences, or the tendency to provide socially desirable responses, although responses were de-identified. Furthermore, the extent of overlap between respondents in the present survey and the previous questionnaire [15] could not be determined, because respondent-level data from the previous questionnaire were not available for comparison. In addition, acute and subacute TBAD were combined in the assessment of high-risk imaging features as potential indications for TEVAR, and phase-specific responses could not be determined from the available questionnaire data. The reported thresholds for rapid aortic expansion were not mutually exclusive, as multiple responses were permitted, and imaging intervals and measurement methods were not standardized. Therefore, comparisons between the reported thresholds should be interpreted with caution. Although the questionnaire was tested for technical functionality in Castor, no formal content pilot testing, cognitive interviewing, or assessment of item comprehension was performed. Therefore, differences in individual interpretation of questionnaire items may have affected the consistency of responses. Furthermore, the relatively small sample size and uneven country representation limit the generalizability of the findings and precluded meaningful between-country comparisons, as subgroup sizes were insufficient for formal analysis. Some centers were represented by more than one PI, potentially introducing clustering of responses and reducing the independence of responses. This was addressed through center-weighted sensitivity and within-center concordance analyses. However, residual effects of within-center clustering cannot be fully excluded. Respondents were PIs of an ongoing international randomized trial and may therefore represent clinicians with specific expertise and interest in aortic disease; consequently, the observed variability may underestimate variation in the broader vascular community.

Additionally, the study relied on self-reported data regarding definitions and treatment criteria, which may limit external validity and may not fully reflect actual clinical practice. Responses to hypothetical scenarios may not always correspond to decision-making in real-world clinical situations.

Nevertheless, the high response rate among experienced investigators and clinicians from multiple countries provides an overview of current expert opinion within the SUNDAY collaborative network.

## 5. Conclusions

This survey among SUNDAY trial PIs demonstrated relatively consistent interpretation of the criteria used to distinguish uncomplicated from complicated TBAD, with rupture and clinical signs of malperfusion consistently recognized as indications for TEVAR. In contrast, variation was observed in the interpretation of high-risk imaging features, definitions of rapid aortic expansion, and clinical criteria such as refractory pain and hypertension. Furthermore, reported preferences for TEVAR treatment strategies varied, including procedural approaches and the use of adjunctive techniques.

These findings may reflect ongoing uncertainty in decision-making regarding the management of uTBAD within the SUNDAY trial network, related to limited evidence, heterogeneous guideline recommendations, and inconsistent definitions of key clinical features. Ongoing prospective trials, including SUNDAY, IMPROVE-AD, and EARNEST, are needed to determine which patients benefit from TEVAR and to further define the role of intervention in uTBAD.

## Figures and Tables

**Table 1:** Demographics per country.

	Total (*n* = 43)	Denmark (*n* = 4)	Finland (*n* = 5)	Hungary (*n* = 1)	New Zealand (*n* = 2)	Norway (*n* = 2)	Sweden (*n* = 13)	The Netherlands (*n* = 16)
**Variables, No. (%)**								
Total population	43 (100%)	4 (9.3%)	5 (11.6%)	1 (2.3%)	2 (4.7%)	2 (4.7%)	13 (30.2%)	16 (37.2%)
Participating centers of origin	31 (100%)	4 (12.9%)	5 (16.1%)	1 (3.2%)	2 (6.5%)	2 (6.5%)	7 (22.6%)	10 (32.3%)
Profession								
Cardiac surgeon	1 (2.3%)	0 (0%)	1 (20%)	0 (0%)	0 (0%)	0 (0%)	0 (0%)	0 (0%)
Interventional radiologist	2 (4.7%)	0 (0%)	2 (40%)	0 (0%)	0 (0%)	0 (0%)	0 (0%)	0 (0%)
Vascular surgeon	40 (93%)	4 (100%)	2 (40%)	1 (100%)	2 (100%)	2 (100%)	13 (100%)	16 (100%)
Local dissection protocol available	37 (86%)	3 (75%)	4 (80%)	0 (0%)	1 (50%)	0 (0%)	13 (100%)	16 (100%)
Dedicated aortic team available	41 (95.3%)	4 (100%)	5 (100%)	1 (100%)	2 (100%)	2 (100%)	11 (84.6%)	16 (100%)
**Variables, mean (st. deviation)**								
Years of working experience	17.4 (7.5)	23.8 (8.5)	18.2 (10.1)	28 (-)	10.0 (0.0)	23.5 (3.5)	17.5 (5.8)	14.8 (7.0)
**Variables, median (IQR 0.25–0.75)**								
Numbers of uTBAD * treated per year per hospital	15 (10)	12.5 (9)	8 (14)	8 (-)	26 (-)	9 (-)	12 (14)	17.5 (5)

* uTBAD: uncomplicated type B aortic dissection.

**Table 2:** Respondents’ perceived definition of rapid aortic expansion.

Variables, No. (%)	Total (*n* = 43)	Denmark (*n* = 4)	Finland (*n* = 5)	Hungary (*n* = 1)	New Zealand (*n* = 2)	Norway (*n* = 2)	Sweden (*n* = 13)	The Netherlands (*n* = 16)
Definition of Rapid Aortic Expansion								
>5 mm in acute phase	21 (48.8%)	1 (25%)	4 (80%)	0 (0%)	0 (0%)	2 (100%)	5 (38.5%)	9 (56.3%)
>10 mm in acute phase	25 (58.1%)	2 (50%)	1 (20%)	1 (100%)	2 (100%)	1 (50%)	9 (69.2%)	9 (56.3%)
>5 mm in subacute phase	7 (16.3%)	1 (25%)	2 (40%)	0 (0%)	0 (0%)	0 (0%)	2 (15.4%)	2 (12.5%)
>10 mm in subacute phase	22 (51.2%)	1 (25%)	2 (40%)	1 (100%)	2 (100%)	1 (50%)	8 (61.5%)	7 (43.8%)
Other *	1 (2.3%)	0 (0%)	0 (0%)	0 (0%)	0 (0%)	0 (0%)	0 (0%)	1 (6.3%)

* Other: we do not have a proper definition. Multiple responses were allowed; percentages therefore do not necessarily sum to 100%.

## Data Availability

All data collected for this study are available upon reasonable request. Requests for access to the data will be reviewed by the corresponding author and the study supervisor. Data will be shared where appropriate and in accordance with applicable ethical and privacy requirements.

## Supplementary Materials

The following supporting information can be downloaded at https://www.mdpi.com/article/10.3390/jcm15197604/s1, Supplementary Material S1: Checklist for Reporting of Survey Studies (CROSS); Supplementary Material S2: List of SUNDAY collaborators by region and hospital; Supplementary Material S3: Survey: TEVAR in uncomplicated type B aortic dissection; Supplementary Material S4: Complete questionnaire results for the full respondent population, including the center-weighted sensitivity analysis and within-center concordance analysis; Table S1: Definitions of complicated TBAD and rapid aortic expansion: respondent-level and center-weighted results; Table S2: TEVAR indications in uTBAD: respondent-level and center-weighted results; Table S3: TEVAR treatment strategies: respondent-level and center-weighted results; Table S4: Within-center concordance in definitions of complicated TBAD and rapid aortic expansion; Table S5: Within-center concordance in perceived TEVAR indications; Table S6: Within-center concordance in TEVAR treatment strategies.

## Abbreviations

The following abbreviations are used in this manuscript:

AAA	Abdominal aortic aneurysm
CABG	Coronary artery bypass grafting
CSF	Cerebrospinal fluid
EARNEST	Early Aortic Repair in patients Needing Endovascular/open Surgery for Type B Aortic Dissection
ESVS	European Society for Vascular Surgery
IMH	Intramural hematoma
IMPROVE-AD	IMPRoving Outcomes in Vascular DisEase—Aortic Dissection
LSA	Left subclavian artery
PI	Principal investigator
SMT	Standard medical therapy
SUNDAY	Scandinavian trial of Uncomplicated Aortic Dissection Therapy
TBAD	Type B aortic dissection
cTBAD	Complicated type B aortic dissection
uTBAD	Uncomplicated type B aortic dissection
TEVAR	Thoracic endovascular aortic repair
