# Supplementary material for: Perspectives on Thoracic Endovascular Aortic Repair for Uncomplicated Type B Aortic Dissection: An International Survey Within the SUNDAY Network
Source: J Clin Med. 2026 Sep 30;15(19):7604. doi: 10.3390/jcm15197604 (PMC13666122; doi:10.3390/jcm15197604)
Supplement: Supplementary file 1 [file jcm-15-07604-s001.zip › Supplementary Material S4.pdf]

Supplementary Material S4

Complete questionnaire results for the full respondent population, including the center-weighted sensitivity analysis and within-center concordance analysis.

Center-weighted analysis
The center-weighted analysis gives equal weight to each participating center, irrespective of the number of respondents per center. Percentages therefore represent the center-weighted proportion across all 31 participating centers.

Definitions

**Variable**	Respondents (*n*=43), n (%)	Center-weighted mean, (*n*=31), %
**Consideration of TBAD complicated in different cases**		
Rupture	43 (100%)	100%
Hypotension/shock	29 (67.4%)	69.4%
Clinical signs malperfusion	43 (100%)	100%
Rapid aortic expansion	34 (79.1%)	79.0%
Refractory hypertension	22 (51.2%)	46.8%
Refractory pain	25 (58.1%)	58.1%
High risk imaging findings	4 (9.3%)	8.1%
**Definition of rapid aortic expansion**		
>5mm in acute phase	21 (48.8%)	50%
>10mm in acute phase	25 (58.1%)	56.5%
>5mm in subacute phase	7 (16.3%)	16.1%
>10mm in subacute phase	22 (51.2%)	53.2%
Other	1 (2.3%)	3.2%

Table S1: Definitions of complicated TBAD and rapid aortic expansion: respondent-level and center-weighted results.

Indications for TEVAR

**Variable**	Respondents (*n*=43), *n* (%)	Center-weighted mean, (*n*=31), %
**TEVAR indication: high-risk imaging features**		
Aortic diameter >40mm	17 (39.5%)	37.1%
False lumen >22mm	6 (14%)	12.9%
Increase >5mm	19 (44.2%)	45.2%
Entry tear >10mm	10 (23.3%)	24.2%
Entry tear <20mm from LSA	1 (2.3%)	1.6%
Entry tear on lesser curvature	4 (9.3%)	9.7%
Bloody pleural effusion	25 (58.1%)	54.8%
Non-bloody pleural effusion	3 (7%)	6.5%
Imaging-only malperfusion	11 (25.6%)	24.2%
Don't rely on high risk features on imaging	16 (37.2%)	37.1%
**Perceived TEVAR indications: complications**		
Rupture	43 (100%)	100%
Hypotension/shock	36 (83.7%)	85.5%
Clinical signs malperfusion	43 (100%)	100%
Refractory hypertension	26 (60.5%)	58.1%
Refractory pain	28 (65.1%)	67.8%
Rapid aortic expansion	38 (88.4%)	88.7%
**Duration of maximal antihypertensive treatment when refractory hypertension is considered an indication for TEVAR**	*n*=26	*n*=21
24 hours	2 (7.7%)	9.5%
48 hours	2 (7.7%)	7.1%
72 hours	6 (23.1%)	21.4%
1 week	7 (26.9%)	26.2%
>1 week	10 (38.5%)	40.5%
**Maximum number of antihypertensive agents when refractory hypertension is considered an indication for TEVAR**	*n*=26	*n*=21
Not applicable	8 (30.8%)	31.0%
>2	0 (0%)	0%
>3	7 (26.9%)	26.2%
>4	3 (11.5%)	11.9%
>5	8 (30.8%)	31.0%
**Duration of maximal analgesia when refractory pain is considered an indication for TEVAR**	*n*=28	*n*=23
Not applicable	2 (7.1%)	6.5%
>12 hours	3 (10.7%)	10.9%
>24 hours	6 (21.4%)	17.4%
>48 hours	9 (32.1%)	32.6%
>1 week	8 (28.6%)	32.6%

**Indication for TEVAR in clinical practice**		
At least one high risk imaging finding	4 (9.3%)	9.7%
Multiple high risk findings	16 (37.2%)	38.7%
Development of clinical complications	37 (86%)	87.1%
No TEVAR in uTBAD	9 (20.9%)	21.0%

Table S2: TEVAR indications in uTBAD: respondent-level and center-weighted results.

TEVAR treatment

**Variable**	Respondents (*n*=43), *n* (%)	Center-weighted mean, (*n*=31), %
**Preferred strategy for stenting**		
Spot stenting	25 (58.1%)	51.6%
All (re-)entry tears in descending aorta	4 (9.3%)	9.7%
Entry tear + descendens until celiac trunk	8 (18.6%)	24.2%
Entry tear + total diseased aorta	3 (7.0%)	8.1%
Entry tear + rest with Petticoat/Stabilize	3 (7.0%)	6.5%
TEVAR + False lumen occlusion (FLO)	0 (0%)	0%
**Consideration of healthy proximal landing zone**		
No intramural hematoma (IMH) or dissection	34 (79.1%)	79.0%
Only IMH	8 (18.6%)	19.4%
IMH or dissection not considered if within IFU	1 (2.3%)	1.6%
**Preferred healthy landing zone**		
0 cm	1 (2.3%)	1.6%
1 cm	2 (4.7%)	3.2%
1.1 cm	1 (2.3%)	1.6%
1.5 cm	2 (4.7%)	4.8%
2 cm	25 (58.1%)	56.5%
3 cm	12 (27.9%)	32.3%
**Minimal healthy landing zone**		
0 cm	0 (0%)	0%
1 cm	13 (30.2%)	27.4%
1.5 cm	2 (4.7%)	3.2%
2 cm	27 (62.8%)	66.1%
3 cm	1 (2.3%)	3.2%
**Preferred sizing/oversizing (in %)**		
0%	3 (7.0%)	4.8%
3%	1 (2.3%)	1.6%
5%	10 (23.3%)	19.4%
7%	1 (2.3%)	1.6%
10%	22 (51.2%)	53.2%
12.5%	1 (2.3%)	3.2%
15%	4 (9.3%)	12.9%
20%	1 (2.3%)	3.2%
**Proximal diameter sizing based on**		
Actual proximal sealing zone	23 (53.5%)	56.5%
Unaffected aorta	21 (48.8%)	45.2%
**Coverage from LSA take-off for zone 4/5 entry tear**		
Yes, regardless of proximal pathology	4 (9.3%)	11.3%
Yes, only in presence of proximal pathology	25 (58.1%)	59.7%
No, only entry tear	14 (32.6%)	29.0%

**Timing elective LSA revascularization**		
Always preventively	25 (58.1%)	56.5%
In case of symptoms	6 (14%)	19.4%
Based on anatomic features	12 (27.9%)	24.2%
**Elective LSA revascularization is preferably performed by**		
Carotid-subclavian bypass	15 (34.9%)	45.2%
LSA transposition	0 (0%)	0%
In situ fenestration	2 (4.7%)	3.2%
Off the shelf branched graft	22 (51.2%)	45.2%
Chimney stent	1 (2.3%)	1.6%
Other	3 (7%)	4.8%
**Cerebrospinal fluid (CSF) drainage performed**		
Prophylactic before elective TEVAR	0 (0%)	0%
Aortic coverage >20cm	9 (20.9%)	21.0%
Previous AAA repair	3 (7%)	6.5%
Coverage within 2cm of CA	0 (0%)	0%
Rescue method	37 (86%)	85.5%
**General or local anesthesia**		
General	36 (83.7%)	85.5%
Local	7 (16.3%)	14.5%
**Preferred access route for TEVAR**		
Percutaneous groin	40 (93%)	93.6%
Open groin	1 (2.3%)	3.2%
Open groin for sheath above…	2 (4.7%)	3.2%
**Access site for needed upper extremity access with 5Fr sheath**		
Radial	17 (39.5%)	41.9%
Brachial elbow open	3 (7%)	4.8%
Brachial elbow percutaneous	14 (32.6%)	32.3%
Brachial upper arm open	3 (7%)	6.5%
Brachial upper arm percutaneous	3 (7%)	8.1%
Axillary open	3 (7%)	6.5%
Axillary percutaneous	0 (0%)	0%
**Access site for needed upper extremity access with 12Fr sheath**		
Radial	0 (0%)	0%
Brachial elbow open	3 (7%)	6.5%
Brachial elbow percutaneous	1 (2.3%)	1.6%
Brachial upper arm open	10 (23.3%)	22.6%
Brachial upper arm percutaneous	6 (14%)	16.1%
Axillary open	16 (37.2%)	40.3%
Axillary percutaneous	7 (16.3%)	12.9%

Table S3: TEVAR treatment strategies: respondent-level and center-weighted results.

Within-center concordance

Within-center concordance was assessed among the 12 centers with two responding PIs and represents the proportion of these centers in which both respondents provided the same responses.

Definitions

**Variable**	Within-center concordance, n/N (%)
**Consideration of TBAD complicated in different cases**	
Rupture	12/12 (100%)
Hypotension/shock	5/12 (41.7%)
Clinical signs malperfusion	12/12 (100%)
Rapid aortic expansion	9/12 (75%)
Refractory hypertension	7/12 (58.3%)
Refractory pain	8/12 (66.7%)
High risk imaging findings	9/12 (75%)
**Definition of rapid aortic expansion**	
>5mm in acute phase	9/12 (75%)
>10mm in acute phase	9/12 (75%)
>5mm in subacute phase	10/12 (83.3%)
>10mm in subacute phase	5/12 (41.7%)

Table S4: Within-center concordance in definitions of complicated TBAD and rapid aortic expansion.

Indications for TEVAR

Variable	Within-center concordance, n/N (%)
**TEVAR indication: high-risk imaging features**	
Aortic diameter >40mm	7/12 (58.3%)
False lumen >22mm	8/12 (66.7%)
Increase >5mm	10/12 (83.3%)
Entry tear >10mm	9/12 (75%)
Entry tear <20mm from LSA	11/12 (91.7%)
Entry tear on lesser curvature	10/12 (83.3%)
Bloody pleural effusion	6/12 (50%)
Non-bloody pleural effusion	12/12 (100%)
Imaging-only malperfusion	7/12 (58.3%)
Don't rely on high risk features on imaging	9/12 (75%)
**Perceived TEVAR indications: complications**	
Rupture	12/12 (100%)
Hypotension/shock	7/12 (58.3%)
Clinical signs malperfusion	12/12 (100%)
Refractory hypertension	6/12 (50%)
Refractory pain	8/12 (66.7%)
Rapid aortic expansion	9/12 (75%)
**Duration of maximal antihypertensive treatment when refractory hypertension is considered an indication for TEVAR***	
24 hours	5/5 (100%)
48 hours	4/5 (80%)
72 hours	2/5 (40%)
1 week	2/5 (40%)
>1 week	2/5 (40%)
**Maximum number of antihypertensive agents when refractory hypertension is considered an indication for TEVAR***	2/5 (40%)
**Duration of maximal analgesia when refractory pain is considered an indication for TEVAR***	1/5 (20%)
**Indication for TEVAR in clinical practice**	
At least one high risk imaging finding	10/12 (83.3%)
Multiple high risk findings	8/12 (66.7%)
Development of clinical complications	8/12 (66.7%)
No TEVAR in uTBAD	7/12 (58.3%)

Table S5: Within-center concordance in perceived TEVAR indications.
*Not all participants had to answer these questions; only if they stated that refractory hypertension or pain would be considered indications for TEVAR. Therefore, N=5 in stead of 12.

TEVAR treatment

**Variable**	Within-center concordance, n/N (%)
**Preferred strategy for stenting**	6/12 (50%)
**Consideration of healthy proximal landing zone**	11/12 (91.7%)
**Preferred healthy landing zone**	3/12 (25%)
**Minimal healthy landing zone**	5/12 (41.7%)
**Preferred sizing/oversizing (in %)**	7/12 (58.3%)
**Proximal diameter sizing based on**	
Actual proximal sealing zone	7/12 (58.3%)
Unaffected aorta	6/12 (50%)
**Coverage from LSA take-off for zone 4/5 entry tear**	7/12 (58.3%)
**Timing elective LSA revascularization**	7/12 (58.3%)
**Elective LSA revascularization is preferably performed by**	6/12 (50%)
**Cerebrospinal fluid (CSF) drainage performed**	
Prophylactic before elective TEVAR	12/12 (100%)
Aortic coverage >20cm	9/12 (75%)
Previous AAA repair	10/12 (83.3%)
Coverage within 2cm of CA	12/12 (100%)
Rescue method	11/12 (91.7%)
**General or local anesthesia**	11/12 (91.7%)
**Preferred access route for TEVAR**	12/12 (100%)
**Access site for needed upper extremity access with 5Fr sheath**	8/12 (66.7%)
**Access site for needed upper extremity access with 12Fr sheath**	6/12 (50%)

Table S6: Within-center concordance in TEVAR treatment strategies.
