# Supplementary material for: Perspectives on Thoracic Endovascular Aortic Repair for Uncomplicated Type B Aortic Dissection: An International Survey Within the SUNDAY Network
Source: J Clin Med. 2026 Sep 30;15(19):7604. doi: 10.3390/jcm15197604 (PMC13666122; doi:10.3390/jcm15197604)
Supplement: Supplementary file 1 [file jcm-15-07604-s001.zip › Supplementary Material S3.pdf]

Supplementary Material S3

Survey: TEVAR in uncomplicated type B aortic dissection

Introduction

This survey aims to investigate potential variations in local practice regarding TEVAR-treatment of uncomplicated type B aortic dissections (uTBAD). The survey is divided into several topics.

1. Definitions: a series of questions to assess your definitions of certain terms used in the indication for and treatment of type B aortic dissection (TBAD) with TEVAR; when do you consider a TBAD to be complicated?
2. Indication for TEVAR: at what point do you consider these complications of TBAD to be an indication for TEVAR?
3. TEVAR-treatment: questions regarding techniques of TEVAR and additional/preventive/prophylactic procedures in uTBAD. This refers to the subacute phase, since we are interested in the treatment after uTBAD.

<u>Baseline/demographics</u>

1. Country
2. Discipline
3. Years of working experience
4. How many uTBAD cases are treated per year in your hospital?
5. Is there a local protocol available for dissections in your hospital?
6. Is there a dedicated aortic team available in your hospital?

<u>Definitions</u>

1. In which case(s) do you assess a TBAD to be complicated? (multiple answers possible)
    a. Rupture
    b. Hypotension/shock
    c. Clinical signs of malperfusion
    d. Rapid aortic expansion
    e. Refractory hypertension
    f. Refractory pain
    g. "High risk" imaging findings as stated in the EACTS guideline, such as *(open ended question)*

2. How do you define rapid aortic expansion in the follow-up of an uTBAD? (multiple answers possible)
    a. Increase in total aortic diameter of >5mm between serial imaging studies in the acute phase (0-14 days)

b. Increase in total aortic diameter of >10mm in the acute phase (0-14 days)
c. Increase in total aortic diameter of >5mm in the subacute phase (14-90 days)
d. Increase in total aortic diameter of >10mm in the subacute phase (14-90 days)
e. Other

<u>Indication for TEVAR</u>

1. Which "high risk" imaging findings, as stated in the EACTS and ACC/AHA guidelines, do you consider to be a potential indication for TEVAR in uTBAD in the acute and subacute phase? (multiple answers possible)
    a. Total aortic diameter >40mm
    b. False lumen diameter >22mm
    c. Increase in total aortic diameter of >5mm between serial imaging studies
    d. Entry tear >10mm
    e. Primary entry tear located <20mm in relation to the left subclavian artery (LSA)
    f. Entry tear on the lesser curvature
    g. Bloody pleural effusion
    h. Non-bloody pleural effusion
    i. Imaging-only evidence of malperfusion
    j. We don't rely on high risk features in clinical practice for decision making

2. Which of the clinically complicated features do you consider to be a potential indication for TEVAR in complicated TBAD? (multiple answers possible)
    a. Rupture
    b. Hypotension or shock
    c. Clinical signs of malperfusion
    d. Refractory hypertension
    e. Refractory pain
    f. Rapid aortic expansion

In case of "D" for question 2:

3. After what duration of maximal antihypertensive therapy without reaching treatment goals, do you consider TEVAR to be indicated in uTBAD?
    a. 24 hours
    b. 48 hours
    c. 72 hours
    d. 1 week
    e. >1 week

In case of "D" for question 2:

4. What do you consider to be the maximum number of maximally dosed antihypertensive agents after which you consider TEVAR to be indicated in uTBAD?
    a. Not applicable
    b. >2
    c. >3
    d. >4
    e. >5

In case of "E" at question 2:

5. After what duration of refractory pain, despite adequate blood pressure control, do you consider TEVAR to be indicated in uTBAD?
    a. Not applicable
    b. >12 hours of pain on maximum analgesia
    c. >24 hours of pain on maximum analgesia
    d. >48 hours of pain on maximum analgesia
    e. >1 week of pain on maximum analgesia

6. In clinical practice, what criteria do you use to decide to perform TEVAR in patients with uTBAD?
    a. Presence of at least one high risk imaging finding
    b. Presence of multiple high risk imaging findings
    c. Development of clinical complications after initially uncomplicated TBAD
    d. We typically don't perform TEVAR in uTBAD

<u>TEVAR-treatment (in uTBAD)</u>

1. What is your preferred strategy for stenting (if possible) in uTBAD?
    a. Covering the proximal entry tear (spot stenting)
    b. Covering all (re-)entry tears in the descending aorta
    c. Covering the entry tear and the entire thoracic descending aorta until the celiac trunk with a stentgraft
    d. Covering the entry tear + the total length of the diseased aorta
    e. Covering the primary entry tear with a stentgraft and the rest of the dissected aorta with Petticoat/stabilize
    f. TEVAR and false lumen occlusion (FLO)

2. What do you consider to be a healthy proximal landing zone?
    a. No IMH or dissection present
    b. Only IMH
    c. IMH or dissection does not play a role as long as the diameter and length is within range of instructions for use (IFU)

3. What do you consider to be the preferred length of the healthy proximal landing zone (including presence of IMH if you think this is ok) for TEVAR?
    a. *Open-ended question (rounded to whole centimeters)*

4. What do you consider should be the minimal length of the "healthy proximal landing zone" for TEVAR in uTBAD?
    a. *Open-ended question (rounded to whole centimeters)*

5. What do you consider should be the preferred diameter sizing/oversizing?
    a. *Open-ended question (percentages rounded to whole numbers)*

6. On which zone do you base your proximal diameter sizing? (multiple answers possible)
    a. Actual proximal sealing zone (affected or unaffected)
    b. Sizing on unaffected aorta: sealing zone if unaffected or proximal to intended sealing zone if sealing zone is affected

7. In cases of uTBAD with entry tear in zone 4 or zone 5, do you as a rule prefer to cover the aorta from the left subclavian artery take-off, regardless of the presence or absence of proximal pathology?
    a. Yes, regardless of the presence or absence of proximal pathology
    b. Yes, only in the presence of proximal pathology
    c. No, only covering the entry tear

8. In case of coverage of the LSA by TEVAR, revascularization of LSA is:
    a. Always preventively performed (in 2 separate surgeries or during same surgery)
    b. Only performed in case of symptoms after TEVAR
    c. Based on anatomic features such as: *(open-ended option)*

9. Revascularization of LSA is preferably performed by:
    a. Carotid-subclavian bypass
    b. LSA transposition
    c. In situ fenestration
    d. Off the shelf branched graft
    e. Chimney stent
    f. Other: *(open ended question)*

10. Cerebrospinal fluid (CSF) drainage is performed (multiple answers possible):
    a. Always as prophylactic procedure before elective TEVAR
    b. In case of extensive thoracic aortic coverage (>20cm)
    c. In case of previous AAA repair
    d. In case of coverage within 2cm of the celiac artery
    e. Only as rescue method in case of symptoms post-TEVAR

11. Do you prefer general or local anesthesia?
    a. General anesthesia
    b. Local anesthesia

12. What is your preferred access route for TEVAR?
    a. Percutaneous groin access
    b. Open groin access
    c. Open groin access for sheath sizes over … Fr; (*open ended, in Fr)*

13. In case of upper extremity access needed with 5Fr sheath, what is the preferred access site?
    a. Radial
    b. Brachial elbow open
    c. Brachial elbow percutaneous
    d. Brachial upper arm open
    e. Brachial upper arm percutaneous
    f. Axillary open
    g. Axillary percutaneous

14. In case of upper extremity access needed with 12Fr sheath, what is the preferred access site?
    h. Radial
    i. Brachial elbow open
    j. Brachial elbow percutaneous
    k. Brachial upper arm open
    l. Brachial upper arm percutaneous
    m. Axillary open
    n. Axillary percutaneous
