# Supplementary material for: Perspectives on Thoracic Endovascular Aortic Repair for Uncomplicated Type B Aortic Dissection: An International Survey Within the SUNDAY Network
Source: J Clin Med. 2026 Sep 30;15(19):7604. doi: 10.3390/jcm15197604 (PMC13666122; doi:10.3390/jcm15197604)
Supplement: Supplementary file 1 [file jcm-15-07604-s001.zip › Supplementary Material S2.pdf]

Supplementary Material S2

List of SUNDAY collaborators by region and hospital

**Denmark**	
- Aarhus University Hospital	Jacob Budtz-Lilly
- Odense University Hospital	Jes Sanddal Lindholt
- Aalborg University Hospital	Christian Nikolaj Petersen
- Copenhagen University/Rigshospitalet	Timothy Resch
**Finland**	
- Helsinki University Hospital	Mikko Jormalainen
- Tampere University Hospital	Velipekka Suominen
- Oulu University Hospital	Juho Kariniemi
	Johanna Herajärvi
	Panu Taskinen
- Kuopio University Hospital	Petri Saari
	Annastiina Husso
- Turku University Hospital	Veikko Nikulainen
**Hungary**	
- Budapest	Zoltán Szeberinz
**Iceland**	
- Landspitali University Hospital	Oscar David Rubio Bemeo
	Elin Hanna Laxdal
**New Zealand**	
- Waikato Hospital	Manar Khashram
- Auckland City Hospital	Anastasia Dean
**Norway**	
- Oslo University Hospital	Eric Dorenberg
	Beate Rikken Lindberg
- St. Olavs Hospital	Martin Ekkehard Altreuthe
- Haukeland University Hospital	Vegard Ellensen
- University Hospital Nord Troms	Knut Eivind Kjørstad
**Sweden**	
- Uppsala University Hospital	Kevin Mani
- Skånes University Hospital	Nuno Dias
	Angelos Karelis

- Norrland University Hospital	Mari Holsti
- Karolinska University Hospital	Rebecka Hultgren
	Magnus Jonsson
- Orebro University Hospital	David McGreevy
	Tal Horer
- Linköping University Hospital	Robert Lind
	Artai Pirouzram
- Sahlgrenska University Hospital	Sofia Strömberg
	Joakim Nordanstig
- Södersjukhset	David Lindström
	Christian Smedberg

The Netherlands

- Leiden University Medical Center	Joost van der Vorst
	Jan van Schaik
- Amsterdam University Medical Center	Kak Khee Yeung
	Vincent Jongkind
- Erasmus Medical Center	Hence Verhagen
	Jorg de Bruin
- Maastricht University Medical Center	Barend Mees
	Geert Willem Schurink
- Radboud University Medical Center	Mark Dirven
	Robin Heijmen
- University Medical Center Groningen	Jean-Paul de Vries
	Ignace Tielliu
- University Medical Center Utrecht	Martin Teraa
	Joost van Herwaarden
- Catharina Hospital Eindhoven	Phillipe Cuypers
- OLVG Amsterdam	Hans Pieter van 't Sant
- Elisabeth-Tweesteden Hospital	Patrick Vriens
- Isala Zwolle	Maurice Pierie
	Olaf Schouten
